# Supplementary material for: Pulmonary Application of Novel Antigen-Loaded Chitosan Nano-Particles Co-Administered with the Mucosal Adjuvant C-Di-AMP Resulted in Enhanced Immune Stimulation and Dose Sparing Capacity
Source: Pharmaceutics. 2023 Apr 13;15(4):1238. doi: 10.3390/pharmaceutics15041238 (PMC10145907; doi:10.3390/pharmaceutics15041238)
Supplement: Supplementary file 1 [file pharmaceutics-15-01238-s001.zip › pharmaceutics-2096937-supplementary.pdf]

Supplementary information:

Single cell suspensions derived from draining LNs (cervical LNs), lung and spleens were stained with Abs for viability (Live/dead), Thy1.1, $CD3^+$, $CD4^+$ and $CD8^+$ and analyzed by flow cytometry. The gating strategy is shown in **Supplementary Figure S1**. The proliferation of Thy1.1 $CD4^+$ (**Fig. 5 A-B**) or $CD8^+$ transferred T cells (**Fig. 5 C-D**) was measured by CFSE dilution (loss of CFSE intensity) and is shown as histograms.

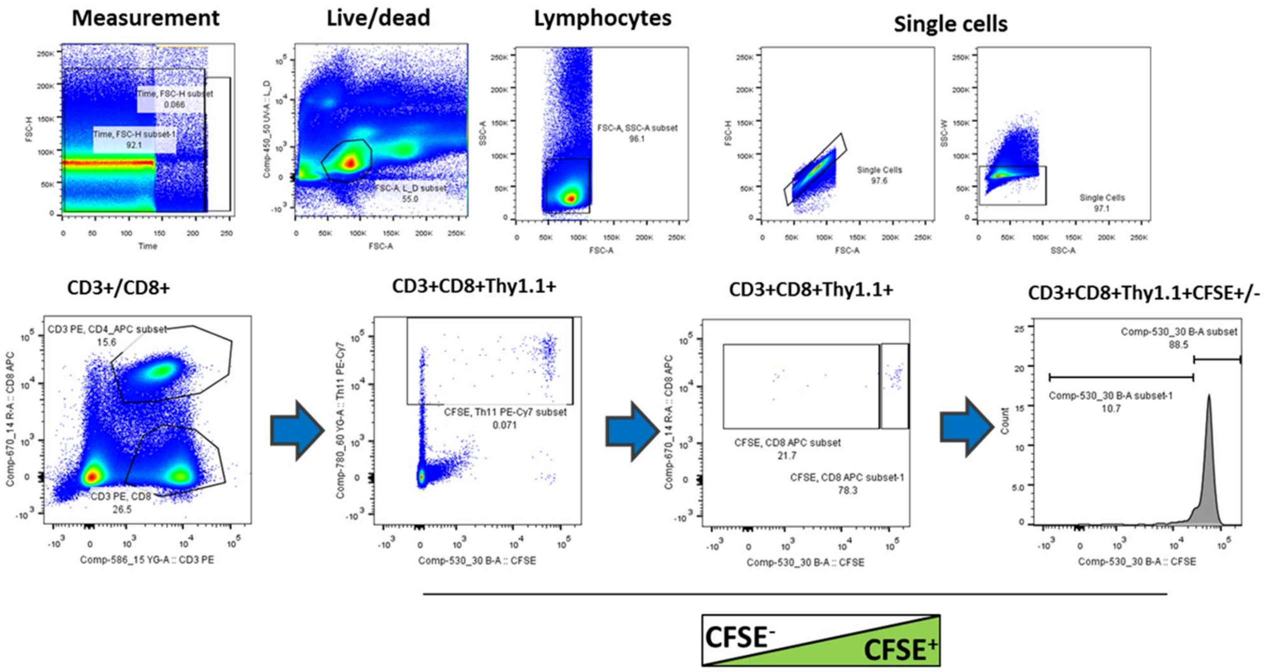

Supplementary Figure S1: Gating strategy of CFSE-stained $Thy1.1^+CD3^+CD8^+$ T cells (OTI).

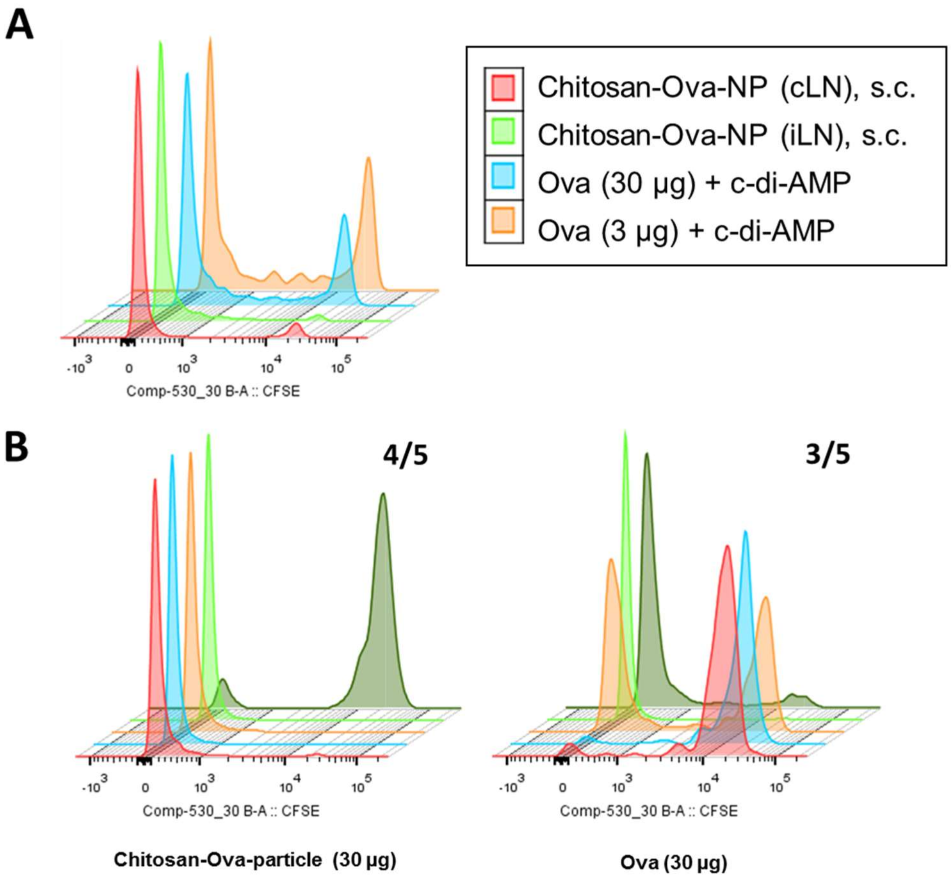

Supplementary Figure S2: A) Dose sparing potency using OVA protein **B).** Comparison of proliferation capacity of OVA protein vs. chitosan-OVA nanoparticles.
